# Anticipating interactions: a predictive diffusion framework for evolving bimanual manipulation

**DOI:** 10.3389/fnbot.2026.1927097

**Published:** 2026-10-06

**Authors:** Xukun Liu, Fengjuan Xie, Xuzhou He, Aifeng Liu, Lijun Yan, Jianwei Yang, Hongsheng Huang, Shenggang Wei

**Affiliations:** 1 Northwest Institute of Mechanical and Electrical Engineering, Xianyang, Shaanxi, China; 2 School of Energy and Power Engineering, Xi'an Jiaotong University, Xi'an, Shaanxi, China

**Keywords:** affordance learning, bimanual manipulation, diffusion policy, Graph Transformer, predictive scene modeling

## Abstract

**Introduction:**

Bimanual robotic manipulation remains a significant challenge in embodied AI, as it requires synchronized motion planning, inter-arm collision avoidance, and dynamic role allocation over long task horizons. Although existing diffusion-based policies and affordance learning methods have demonstrated considerable capability, they largely treat the environment as static and fail to model how interaction relationships and coordination patterns evolve over time.

**Methods:**

This paper introduces the Predictive Affordance and Coordination Evolution Diffusion Policy (PACE-DP), a unified framework that explicitly forecasts future scene dynamics and models the temporal progression of manipulation affordances and bimanual coordination. PACE-DP integrates three predictive components: (1) a Predictive Scene Module that forecasts latent future object-centric representations; (2) an Affordance and Coordination Evolution Module that constructs temporally evolving interaction graphs and employs a Graph Transformer to predict future relation types, grasp success probabilities, coordination modes, and spatial alignment targets; and (3) a Conditional Diffusion Policy that generates smooth, coordinated action sequences conditioned on fused current and predicted features.

**Results:**

We evaluate PACE-DP on the RoboTwin 2.0 platform across 10 diverse bimanual tasks, including handover, placement, stacking, and switch operation. Experimental results show that PACE-DP performs favorably compared with baseline methods, achieving higher success rates on coordination-intensive tasks with only marginal computational overhead.

**Discussion:**

These findings suggest that explicitly predicting future affordance and coordination evolution provides an effective inductive bias for bimanual manipulation, enabling more reliable coordination without substantially increasing computational cost. PACE-DP thus offers a promising step toward predictive, temporally aware policies for long-horizon bimanual tasks.

## 1 Introduction

Bimanual robotic manipulation—the coordination of two arms to perform complex, object-centric tasks—represents a fundamental challenge in embodied artificial intelligence. Unlike single-arm systems, dual-arm manipulation demands synchronized motion planning, inter-arm collision avoidance, and dynamic role allocation between effectors. These challenges are compounded by long-horizon sequencing, spatial uncertainty, and physical contact with diverse objects. Despite significant advances in imitation learning and generative modeling, existing methods struggle with a core difficulty: anticipating how manipulation relationships and coordination patterns evolve over time and adapting actions accordingly.

Diffusion models have recently emerged as a powerful paradigm for robot policy learning, demonstrating superior capability in modeling multimodal action distributions and generating smooth, temporally consistent trajectories. Extensions incorporating 3D representations, acceleration techniques, and large-scale architectures have further improved efficiency and generalization. However, these approaches largely treat the environment as a static configuration and do not explicitly model how the underlying manipulation landscape evolves as tasks unfold. Meanwhile, affordance learning and relational reasoning have provided tools for understanding action feasibility, yet typically operate on static snapshots and fail to capture temporal dynamics of interaction structures.

This paper introduces Predictive Affordance and Coordination Evolution Diffusion Policy (PACE-DP), a unified framework that integrates three predictive capacities into a diffusion-based action generation pipeline. A Predictive Scene Module forecasts latent representations of future scene states. An Affordance and Coordination Evolution Module constructs temporally evolving interaction graphs over scene entities and employs a Graph Transformer architecture to model how manipulation affordances and coordination relations change over time, explicitly predicting future relation types, grasp success probabilities, coordination modes, and spatial alignment targets. A Conditional Diffusion Policy then generates bimanual action sequences conditioned on a fused representation combining current scene features, predicted future states, relation embeddings, and coordination embeddings.

We evaluate PACE-DP on the RoboTwin 2.0 platform across 10 diverse bimanual tasks including handover, placement, stacking, and switch operation. Experimental results demonstrate that our approach outperforms baseline methods, achieving higher success rate improvements on coordination-intensive tasks while incurring only marginal computational overhead.

## 2 Related work

Diffusion models have recently emerged as an effective paradigm for robot policy learning due to their ability to model multimodal action distributions and generate smooth trajectories. Diffusion Policy (DP) (Chi et al., 2025) formulates action generation as an iterative denoising process and demonstrates superior performance over conventional behavior cloning approaches. Subsequent studies extended diffusion models to planning and offline reinforcement learning (Janner et al., 2022; Ajay et al., 2022; Song et al., 2020). To improve perception capability, 3D Diffusion Policy (DP3) (Ze et al., 2024) incorporates point-cloud representations and significantly enhances sample efficiency and generalization. Further developments such as iDP3 (Ze et al., 2025), ManiCM (Lu et al., 2024), OneDP (Wang et al., 2024), and large-scale Transformer-based diffusion policies (Zhu et al., 2025) have improved inference speed, scalability, and robustness. Diffusion Policy Policy Optimization (DPPO) (Ren et al., 2025) further combines diffusion models with policy optimization to enhance long-horizon decision making.

Bimanual manipulation requires synchronized control and interaction reasoning between two arms. Early approaches mainly relied on reinforcement learning and hierarchical policies (Nachum et al., 2018; Haarnoja et al., 2018). With the emergence of foundation models, RT-1 (Brohan et al., 2022) and Gato (Reed et al., 2022) demonstrated scalable robot learning from large datasets. Recent studies have focused on dual-arm coordination. Motoda et al. (2025) proposed transformer-based action chunking with inter-arm coordination for bimanual manipulation. Choice Policies (Qi et al., 2025) introduced coordinated humanoid manipulation through policy switching. Bi-VLA (Gbagbe et al., 2024) integrated vision-language-action models for dexterous bimanual tasks. RoboTwin 2.0 (Chen et al., 2025) provides a large-scale benchmark and synthetic data generation framework for robust dual-arm manipulation, while RoboMIND 2.0 (Hou et al., 2025) extends the scale to multimodal and mobile bimanual tasks.

Understanding object affordances and interaction relationships is essential for complex manipulation. Relational inductive biases and graph neural networks provide powerful tools for modeling structured interactions (Battaglia et al., 2018). Graph Attention Networks (GAT) (Veličković et al., 2017) enable adaptive information aggregation and have been widely adopted in robotics. Recent studies incorporate graph reasoning into manipulation tasks to model object-object and robot-object interactions (Deng et al., 2024; Zhang et al., 2024).

Meanwhile, affordance learning has been explored to estimate graspability and contact feasibility from visual observations (Wu et al., 2023; Wi et al., 2023). However, most existing approaches assume static relationships and fail to capture how affordances and coordination patterns evolve during task execution. Modeling dynamic relationship evolution remains an open challenge for long-horizon bimanual manipulation.

Predictive world models improve decision making by forecasting future states. Classical model-based reinforcement learning methods such as Probabilistic Inference for Learning COntrol (PILCO) (Deisenroth and Rasmussen, 2011) and neural dynamics models (Nagabandi et al., 2018) demonstrate the advantages of learning environment dynamics. Dreamer and DreamerV2 (Hafner et al., 2019, 2020) further employ latent imagination to perform planning in learned representations. Recent embodied AI studies incorporate predictive representations to enhance generalization and long-horizon reasoning (Huang et al., 2022; Fung et al., 2025; Feng et al., 2025). Most predictive models, however, focus on state transitions or latent imagination without explicitly considering the evolution of manipulation relationships and coordination structures.

## 3 Method

This chapter presents Predictive Affordance and Coordination Evolution Diffusion Policy (PACE-DP), a diffusion-based framework for bimanual manipulation that explicitly models future scene dynamics and the evolutionary process of interaction relationships. The framework comprises four core components: a Point Cloud Encoder that constructs geometric and spatial representations from multi-view RGB-D observations; a Predictive Scene Module that forecasts latent future scene states; an Affordance and Coordination Evolution Module that models the temporal dynamics of manipulation relationships and dual-arm coordination patterns via interaction graphs and Graph Transformer architectures; and a Conditional Diffusion Policy that generates action sequences conditioned on fused current, predictive, and relational features (Figure 1).

**Figure 1:**
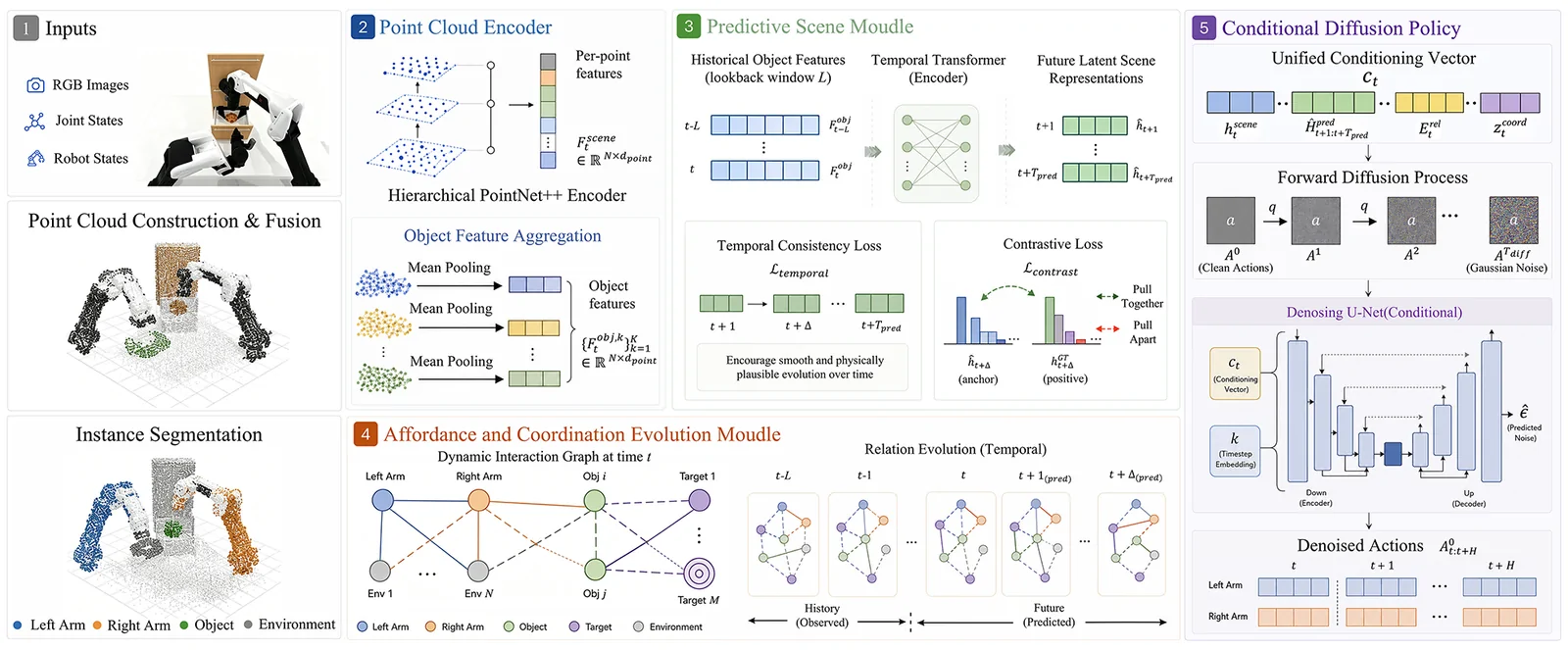
Overall architecture of PACE-DP. Given multi-view RGB-D observations, the Point Cloud Encoder extracts geometric features. The Predictive Scene Module forecasts future latent scene states. The Affordance and Coordination Evolution Module constructs interaction graphs to model relationship evolution and dual-arm coordination. The predicted future embeddings (${\hat{E}}_{t+1:t+T_{pred}}^{rel}$, ${\hat{Z}}_{t+1:t+T_{pred}}^{coord}$) and scene representations (${\hat{H}}_{t+1:t+T_{pred}}^{pred}$) are fused into **c**_*t*_ to condition the diffusion model for action generation.

Formally, let $O_{t}={\left\{I_{t}^{v},D_{t}^{v}\right\}}_{v=1}^{V}\;$denote multi-view RGB-D observations at timestep *t*, where *V* is the number of camera views. The goal is to learn a policy π(**A**_*t*:*t*+*H*_∣**O**_≤ *t*_)that generates a sequence of bimanual actions $A_{t:t+H}=\left\{a_{t}^{\text{l}eft},a_{t}^{\text{r}ight},\ldots ,a_{t+H}^{\text{l}eft},a_{t+H}^{\text{r}ight}\right\}\;$over horizon *H*. PACE-DP integrates three predictive components: future scene forecasting, affordance evolution reasoning, and coordination dynamics modeling. Their predicted outputs are explicitly fed into the diffusion policy as conditioning signals, ensuring actions are directly informed by anticipated future states and relationship changes.

### 3.1 Point cloud representation and encoding

The framework begins by constructing a unified 3D scene representation from multi-view observations. For each view *v*, depth images are projected into 3D point clouds using known camera intrinsics and extrinsics. This yields a point cloud $P_{t}^{v}={\left\{p_{i}\in {\mathbb{R}}^{3}\right\}}_{i=1}^{N_{v}}$ with associated RGB features $c_{i}\in {\mathbb{R}}^{3}$. All view-specific point clouds are transformed into a unified world coordinate system and fused through voxel-based downsampling to obtain a consolidated scene point cloud $P_{t}\in {\mathbb{R}}^{N\times 6}$, where each point encodes both position and color information. Farthest point sampling reduces the point cloud to a fixed size, preserving geometric structure while enabling efficient processing.

A hierarchical PointNet++ style encoder extracts multi-scale geometric features. Let $F_{t}^{\text{scene}}\in {\mathbb{R}}^{N\times d_{\text{point}}}\;$denote the per-point features after passing through Set Abstraction layers:


$$\begin{array}{l} \begin{array}{c} F_{t}^{\text{scene}}=\text{PointNet}++\left(P_{t}\right) \end{array} \end{array}$$ (1)

Instance segmentation masks are obtained using Segment Anything Model (SAM) applied to RGB images, which assign each point an object instance label. We use the ViT-H variant with default parameters provided by the official implementation. Object-level features are aggregated through mean pooling:


$$\begin{array}{l} \begin{array}{c} F_{t}^{\text{obj},k}=\text{MeanPool}\left(\left\{f_{i}\in F_{t}^{\text{scene}}|\left(\text{label}i\right)=k\right\}\right) \end{array} \end{array}$$ (2)

where $F_{t}^{\text{obj},k}\in {\mathbb{R}}^{d_{\text{point}}}$ represents the aggregated feature for the k*k*-th object instance. The set of object features ${\left\{F_{t}^{\text{obj},k}\right\}}_{k=1}^{K}\;$serves as the foundation for both scene prediction and relationship modeling, with *K* denoting the number of objects in the scene.

### 3.2 Predictive scene module

The Predictive Scene Module forecasts latent representations of future scene states without requiring explicit future point cloud reconstruction. This module uses a temporal Transformer architecture that models the evolution of object-centric features over time.

Given historical object features ${\left\{F_{\tau }^{\text{obj}}\right\}}_{\tau =t-L}^{t}$ over a lookback window of length *L*, the features are projected into a latent space:


$$\begin{array}{l} \begin{array}{c} h_{\tau }={\text{MLP}}_{\text{proj}}\left(F_{\tau }^{\text{obj}}\right),h_{\tau }\in {\mathbb{R}}^{K\times d_{\text{latent}}} \end{array} \end{array}$$ (3)

A causal Transformer encoder captures temporal dependencies across objects:


$$\begin{array}{l} \begin{array}{c} H_{t-L:t}={\text{Transformer}}_{\text{scene}}\left(\left\{h_{t-L},\ldots ,h_{t}\right\}\right) \end{array} \end{array}$$ (4)

The future scene representation at horizon Δ∈{1, …, *T*_pred_} is generated via an autoregressive prediction head:


$$\begin{array}{l} \begin{array}{c} {\hat{h}}_{t+\Delta }={\text{MLP}}_{\text{pred}}\left(H_{t},\Delta \right)+\epsilon_{\text{pred}},\epsilon_{\text{pred}}\sim N\left(0,\sigma_{\text{pred}}^{2}\right) \end{array} \end{array}$$ (5)

where the additive noise accounts for stochastic future variations. The predicted latent features ${\left\{{\hat{h}}_{t+\Delta }\right\}}_{\Delta =1}^{T_{\text{pred}}}\;$encode anticipated object positions, orientations, and interaction states without explicit geometric reconstruction.

A temporal consistency loss encourages predicted future representations to maintain temporal smoothness and physical plausibility:


$${ℒ}_{\text{temporal}}=\sum_{T_{\text{pred}}-1}^{\Delta =1}\;∥{\hat{h}}_{t+\Delta +1}-\Phi ({\hat{h}}_{t+\Delta }){∥}_{2}^{2}$$ (6)

where Φ is a learnable transition function implemented as an Multi-Layer Perceptron (MLP). A contrastive objective encourages predicted features to be closer to their corresponding ground-truth future encodings when available from simulator states, while pushing apart predictions for different objects:


$$\begin{array}{l} \begin{array}{c} L_{\text{contrast}}=-\log\frac{\exp\left(\text{sim}\left({\hat{h}}_{t+\Delta },h_{t+\Delta }^{GT}\right)/\tau \right)}{\underset{j\neq i}{\sum }\;\exp\left(\text{sim}\left({\hat{h}}_{t+\Delta },h_{t+\Delta }^{j}\right)/\tau \right)} \end{array} \end{array}$$ (7)

where *sim*(·, ·)denotes cosine similarity and τ is a temperature parameter. Both $L_{\text{temporal}}\;$and $L_{\text{contrast}}\;$provide auxiliary supervision that indirectly benefits the downstream diffusion policy. $L_{\text{temporal}}\;$encourages smooth and physically plausible transitions in the predicted future latent representations, preventing erratic predictions that could misguide action generation. $L_{\text{contrast}}\;$enforces discriminative object-centric features, which facilitates relation reasoning in the Graph Transformer. By improving the quality of the predicted scene representations ${Ĥ}_{t+1:t+T_{\text{pred}}}^{\text{pred}},$ which are later fused into the conditioning vector **c**_*t*_, these auxiliary losses indirectly guide the diffusion policy toward actions better aligned with anticipated future states.

### 3.3 Affordance and coordination evolution module

**Dynamic interaction graph construction**. A temporally evolving interaction graph $G_{t}=\left(V,E_{t}\right)\;$is constructed where vertices V represent all entities in the manipulation scene: left arm $V_{\text{left}}$, right arm ν_right_, each object $V_{\text{obj}}^{k}$, target regions $\nu_{\text{target}}^{m}$, and environmental elements $V_{\text{env}}^{n}\;$such as tabletop surfaces and containers. Edge set $E_{t}\;$captures pairwise relationships at timestep *t*, with each edge $e_{ij}\in E_{t}\;$associated with a relation type $r_{ij}\in R$.

The relation set  Rencompasses both manipulation affordances and coordination patterns. Affordance relations include grasping, contacting, supporting, aligning, and reaching. Coordination relations include co-grasping, handover, co-manipulation, synchronized motion, and role differentiation.

Ground-truth relation types and coordination modes are annotated from simulator states based on inter-entity distance, contact, and relative velocity, following RoboTwin 2.0 (Chen et al., 2025). Specifically, “supporting” is labeled when an object rests on another surface with zero relative velocity; “handover” is labeled when both arms sequentially contact the same object within a short time window; “co-grasping” is labeled when both end-effectors simultaneously maintain contact with the same object. These heuristic rules are applied automatically to simulator states to generate ground-truth labels for training.

**Graph Transformer for relation evolution**. Node features $x_{i}^{\left(t\right)}\in {\mathbb{R}}^{d_{node}}\;$are initialized from the corresponding entity representations: object features use $F_{t}^{\text{obj},k}$; arm features encode joint configurations and end-effector poses; target features encode spatial positions and semantic types. Edge features $e_{ij}^{\left(t\right)}$ are initialized as one-hot encodings of relation types $r_{ij}^{\left(t\right)}$.

The Graph Transformer layer updates node representations through multi-head attention over the graph structure:


$$\begin{array}{l} \begin{array}{c} x_{i}^{\left(t\right),\ell +1}=\text{LayerNorm}\left(x_{i}^{\left(t\right),\ell }+\underset{j\in N\left(i\right)}{\sum }\;\alpha_{ij}^{\ell }W_{V}^{\ell }x_{j}^{\left(t\right),\ell }\right) \end{array} \end{array}$$ (8)

where attention weights $\alpha_{ij}^{l}$ are computed as:


$$\begin{array}{l} \begin{array}{c} \alpha_{ij}^{\ell }=\frac{\exp\left({\left(W_{Q}^{\ell }x_{i}^{\left(t\right),\ell }\right)}^{⊤}\left(W_{K}^{\ell }x_{j}^{\left(t\right),\ell }+W_{E}^{\ell }e_{ij}^{\left(t\right)}\right)/\sqrt{d}\right)}{\underset{k\in N\left(i\right)}{\sum }\;\exp\left({\left(W_{Q}^{\ell }x_{i}^{\left(t\right),\ell }\right)}^{⊤}\left(W_{K}^{\ell }x_{k}^{\left(t\right),\ell }+W_{E}^{\ell }e_{ik}^{\left(t\right)}\right)/\sqrt{d}\right)} \end{array} \end{array}$$ (9)

Temporal evolution is captured by stacking *M* Graph Transformer layers and incorporating temporal positional encodings:


$$\begin{array}{l} \begin{array}{c} {\tilde{x}}_{i}^{\left(t\right)}=x_{i}^{\left(t\right)}+\text{PE}\left(t\right) \end{array} \end{array}$$ (10)

where PE(*t*) denotes sinusoidal positional encoding. The sequence of graph states is processed by a temporal Transformer that predicts future relation embeddings:


$$\begin{array}{l} \begin{array}{c} {\hat{r}}_{ij}^{\left(t+\Delta \right)}={\text{MLP}}_{\text{rel}}\left({\text{Transformer}}_{\text{temp}}{\left(\left\{{\tilde{X}}^{\left(t-L\right)},\ldots ,{\tilde{X}}^{\left(t\right)}\right\}\right)}_{ij},\Delta \right) \end{array} \end{array}$$ (11)

The predicted relation probabilities are obtained via a softmax over R:


$$\hat{p}(r_{ij}^{\left(t+\Delta \right)}=r)=\frac{\exp({\hat{r}}_{ij}^{\left(t+\Delta \right)}[r])}{\sum_{r^{\prime }\in ℛ}\exp({\hat{r}}_{ij}^{\left(t+\Delta \right)}[r^{\prime }])}$$ (12)

**Affordance-aware action feasibility prediction**. Continuous affordance metrics that may inform action generation are predicted for each object-arm pair. Grasp success probability is computed as ${\hat{\gamma }}_{\text{grasp}}^{\left(t+\Delta \right)}=\sigma \left({\text{MLP}}_{\text{grasp}}\left({\hat{x}}_{\text{arm}}^{\left(t+\Delta \right)},{\hat{x}}_{\text{obj}}^{\left(t+\Delta \right)},{\hat{e}}_{\text{arm - obj}}^{\left(t+\Delta \right)}\right)\right)$. Contact force estimation is given by ${\hat{f}}_{\text{contact}}^{\left(t+\Delta \right)}=$ ${\text{MLP}}_{\text{force}}\left({\hat{x}}_{\text{arm}}^{\left(t+\Delta \right)},{\hat{x}}_{obj}^{\left(t+\Delta \right)},{\hat{e}}_{contact}^{\left(t+\Delta \right)}\right)$. The spatial alignment score ${\hat{q}}_{\text{align}}^{\left(t+\Delta \right)}={\text{MLP}}_{\text{align}}\left({\hat{x}}_{\text{obj}}^{\left(t+\Delta \right)},{\hat{x}}_{\text{target}}^{\left(t+\Delta )\right)}\right)\in {\mathbb{R}}^{6}$ represents the desired relative pose. These predicted affordance metrics serve as guidance signals that bias the diffusion policy toward actions compatible with future relationship constraints.

**Bimanual coordination modeling**. A dedicated coordination submodule operates on a bipartite graph between left-arm and right-arm actions. Let $a_{t}^{left}\in {\mathbb{R}}^{d_{a}}$ and $a_{t}^{right}\in {\mathbb{R}}^{d_{a}}\;$denote the action vectors for each arm at timestep *t*, comprising end-effector pose and gripper opening.

A coordination embedding $z_{t}^{\text{coord}}\in {\mathbb{R}}^{d_{\text{coord}}}\;$captures the relative motion pattern:


$$\begin{array}{l} \begin{array}{c} z_{t}^{\text{coord}}={\text{MLP}}_{\text{coord}}\left(\left[a_{t}^{\text{left}};a_{t}^{\text{right}};a_{t-1}^{\text{left}};a_{t-1}^{\text{right}};a_{t}^{\text{left}}-a_{t}^{\text{right}}\right]\right) \end{array} \end{array}$$ (13)

A temporal convolution over a window of coordination embeddings predicts future coordination modes:


$$\begin{array}{l} \begin{array}{c} z_{t:t+H}^{\text{coord}}=\text{TemporalConv}\left(\left\{z_{t-L:t}^{\text{coord}}\right\}\right) \end{array} \end{array}$$ (14)

A coordination classifier predicts the active coordination mode $m_{t+\Delta }\in M_{\text{coord}}\;$at each future timestep:


$$\begin{array}{l} \begin{array}{c} \hat{p}\left(m_{t+\Delta }\right)=\text{Softmax}\left({\text{MLP}}_{\text{mode}}\left(z_{t+\Delta }^{\text{coord}}\right)\right) \end{array} \end{array}$$ (15)

where $M_{\text{coord}}=\left\{\text{independent,sync,handover,cograsp,comanip}\right\}$. This explicit mode prediction allows the policy to anticipate handover transitions and synchronization requirements.

### 3.4 Conditional diffusion policy with integrated features

**Unified conditioning representation**. The features extracted from the Predictive Scene Module and Affordance and Coordination Evolution Module are fused into a unified conditioning vector **c**_*t*_ that guides action generation:


$$\begin{array}{l} \begin{array}{c} c_{t}={\text{MLP}}_{\text{fuse}}\\ \left(\left[h_{t}^{\text{scene}};{\hat{H}}_{t+1:t+T_{\text{pred}}}^{\text{pred}};{\hat{E}}_{t+1:t+T_{\text{pred}}}^{rel};{\hat{Z}}_{t+1:t+T_{\text{pred}}}^{\text{coord}}\right]\right) \end{array} \end{array}$$ (16)

where ${\hat{E}}_{t+1:t+T_{\text{pred}}}^{\text{rel}}$ and ${\hat{Z}}_{t+1:t+T_{\text{pred}}}^{\text{coord}}$ denote the predicted future relation and coordination embeddings from Equations 11–15, respectively. This conditioning vector explicitly incorporates forecasts of how relations and coordination patterns will evolve, rather than relying solely on current state features. The fused conditioning is injected into the denoising network via FiLM layers, ensuring that predicted future information directly modulates action generation at each denoising step.

**Forward pass**. To clarify: at each step t*t*, the Predictive Scene Module forecasts ${\hat{H}}_{t+1:t+T_{\text{pred}}}^{\text{pred}}\;$from historical object features. Concurrently, the Affordance and Coordination Evolution Module predicts future relation embeddings ${\hat{E}}_{t+1:t+T_{\text{pred}}}^{\text{rel}}\;$and coordination embeddings ${\hat{Z}}_{t+1:t+T_{\text{pred}}}^{\text{coord}}\;$via the Graph Transformer. These three predictions are concatenated with current scene encoding $h_{t}^{\text{scene}}\;$to form **c**_*t*_, which conditions the diffusion policy to generate *A*_*t*:*t*+*H*_. Thus, predicted future information—not merely current relations—directly guides action generation.

**Diffusion formulation for bimanual actions**. Action generation is modeled as a denoising process. Let $A_{t:t+H}\in {\mathbb{R}}^{H\times \left(2\cdot d_{a}\right)}\;$denote the concatenated left and right arm action sequence. The forward diffusion process progressively adds Gaussian noise over *T*_diff_ steps:


$$\begin{array}{l} \begin{array}{c} q\left(A^{k}|A^{k-1}\right)=N\left(A^{k};\sqrt{1-\beta_{k}}A^{k-1},\beta_{k}I\right) \end{array} \end{array}$$ (17)

where β_*k*_ is a predefined noise schedule, $A^{0}=A_{t:t+H}$, and $A^{T_{\text{diff}}}\sim N\left(0,I\right)$.

The reverse denoising process recovers the clean action sequence conditioned on **c**_*t*_:


$$\begin{array}{l} \begin{array}{c} p_{\theta }\left(A^{k-1}|A^{k},c_{t}\right)=N\left(A^{k-1};\mu_{\theta }\left(A^{k},c_{t},k\right),\sigma_{k}^{2}I\right) \end{array} \end{array}$$ (18)

A noise prediction network ϵ_θ_ is trained to predict the noise added at each diffusion step:


$$\begin{array}{l} \begin{array}{c} L_{\text{diff}}=E_{k\left[1,T_{\text{diff}}\right],\epsilon \sim N\left(0,I\right),\left(A^{0},c_{t}\right)}\left[∥\epsilon -\epsilon_{\theta }\left(A^{k},c_{t},k\right){∥}_{2}^{2}\right] \end{array} \end{array}$$ (19)

**Denoising network architecture**. The noise prediction network ϵ_θ_ adopts a conditional U-Net architecture adapted for action sequence denoising. The network takes as input the noisy action sequence **A**^*k*^, conditioning vector **c**_*t*_, and diffusion timestep *k*. Feature-wise Linear Modulation (FiLM) layers inject conditioning information:


$$\begin{array}{l} \begin{array}{c} \text{FiLM}\left(x|c_{t}\right)=\gamma \left(c_{t}\right)⊙x+\beta \left(c_{t}\right) \end{array} \end{array}$$ (20)

where γ(·) and β(·) are MLPs that produce modulation parameters. The timestep *k* is encoded via sinusoidal positional embedding followed by an MLP, then added to the feature maps at each resolution level. The network outputs the predicted noise $\hat{\epsilon }$, which is used to compute the denoised action sequence via the Denoising Diffusion Implicit Model (DDIM) sampler.

**Temporal consistency and coordination constraints**. The diffusion loss is augmented with three regularization terms that encourage temporal smoothness and bimanual coordination.

The temporal smoothness loss penalizes abrupt changes in consecutive actions:


$${ℒ}_{\text{smooth}}=\frac{1}{H-1}\sum_{H-1}^{i=1}\left(∥a_{t+i}^{\text{left}}-a_{t+i-1}^{\text{left}}{∥}_{2}^{2}+∥a_{t+i}^{\text{right}}-a_{t+i-1}^{\text{right}}{∥}_{2}^{2}\right)$$ (21)

The coordination consistency loss enforces alignment between generated actions and predicted coordination modes:


$${ℒ}_{\text{coord}}=-\sum_{H-1}^{\Delta =0}\log\hat{\;p}(m_{t+\Delta }|a_{t+\Delta }^{\text{left}},a_{t+\Delta }^{\text{right}})$$ (22)

where $\hat{p}\left(m|a^{\text{left}},a^{\text{right}}\right)$is a lightweight classifier mapping action pairs to coordination modes, trained jointly with the main policy, the negative sign ensures minimization of the negative log-likelihood.

The affordance grounding loss encourages generated actions to be compatible with predicted affordance constraints:


$${ℒ}_{\text{afford}}=\sum_{H-1}^{\Delta =0}\left(-\log{\hat{\gamma }}_{\text{grasp}}^{\left(t+\Delta \right)}\cdot 1_{\text{incontact}}+∥{\hat{q}}_{\text{align}}^{\left(t+\Delta \right)}-q_{r\text{elative}}^{\left(t+\Delta \right)}{∥}_{2}^{2}\right)$$ (23)

The total training objective combines these components:


$$\begin{array}{l} \begin{array}{c} L_{\text{total}}=L_{\text{diff}}+\lambda_{\text{smooth}}L_{\text{smooth}}+\lambda_{\text{coord}}L_{\text{coord}}+\\ \lambda_{\text{afford}}L_{\text{afford}}+\lambda_{\text{temporal}}L_{\text{temporal}}+\lambda_{\text{contrast}}L_{\text{contrast}} \end{array} \end{array}$$ (24)

The loss weights are set to λ_smooth_ = 0.1, λ_coord_ = 0.5, λ_afford_ = 0.3, λ_temporal_ = 0.1, and λ_contrast_ = 0.05, selected based on preliminary validation experiments.

## 4 Experiments

### 4.1 Experimental setup

**Experimental platform**. We evaluate PACE-DP on the RoboTwin 2.0 platform, which provides a unified simulation environment and a scalable benchmark for bimanual robotic manipulation. Dual-arm evaluations are conducted using Aloha-AgileX platforms, as illustrated in the figure below.

**Data generation**. All expert demonstrations were generated using RoboTwin 2.0's automated task execution pipeline. For each task and each dual-arm robot configuration, we generated 50 demonstration trajectories. All reported results are based on models trained using only the training split. For each baseline method (Pi0, ACT, DP, and DP3), we use official open-source codebases with recommended hyperparameters, and strictly follow the official RoboTwin 2.0 evaluation protocol to ensure fair comparison (Chen et al., 2025). All methods are evaluated on the same set of test episodes within the RoboTwin 2.0 simulation environment to ensure fair comparison.

**Policy training**. We generate 50 expert demonstrations for each dual-arm configuration. The data format includes multi-view RGB observations, joint states, and action sequences.

**Task description**. In our experiments, the robot is required to complete a diverse set of bimanual manipulation tasks under varying initial conditions while avoiding collisions. The key challenges include spatial uncertainty of target objects, long-horizon reasoning, and inter-arm interference during task execution. We select these 10 tasks from the RoboTwin 2.0 benchmark as they represent a diverse range of bimanual coordination requirements, including handover (symmetric role switching), placement and stacking (asymmetric role differentiation), and switch operation (sequential bimanual coordination), thereby providing a comprehensive testbed for evaluating different aspects of bimanual manipulation (Figure 2).

**Figure 2:**
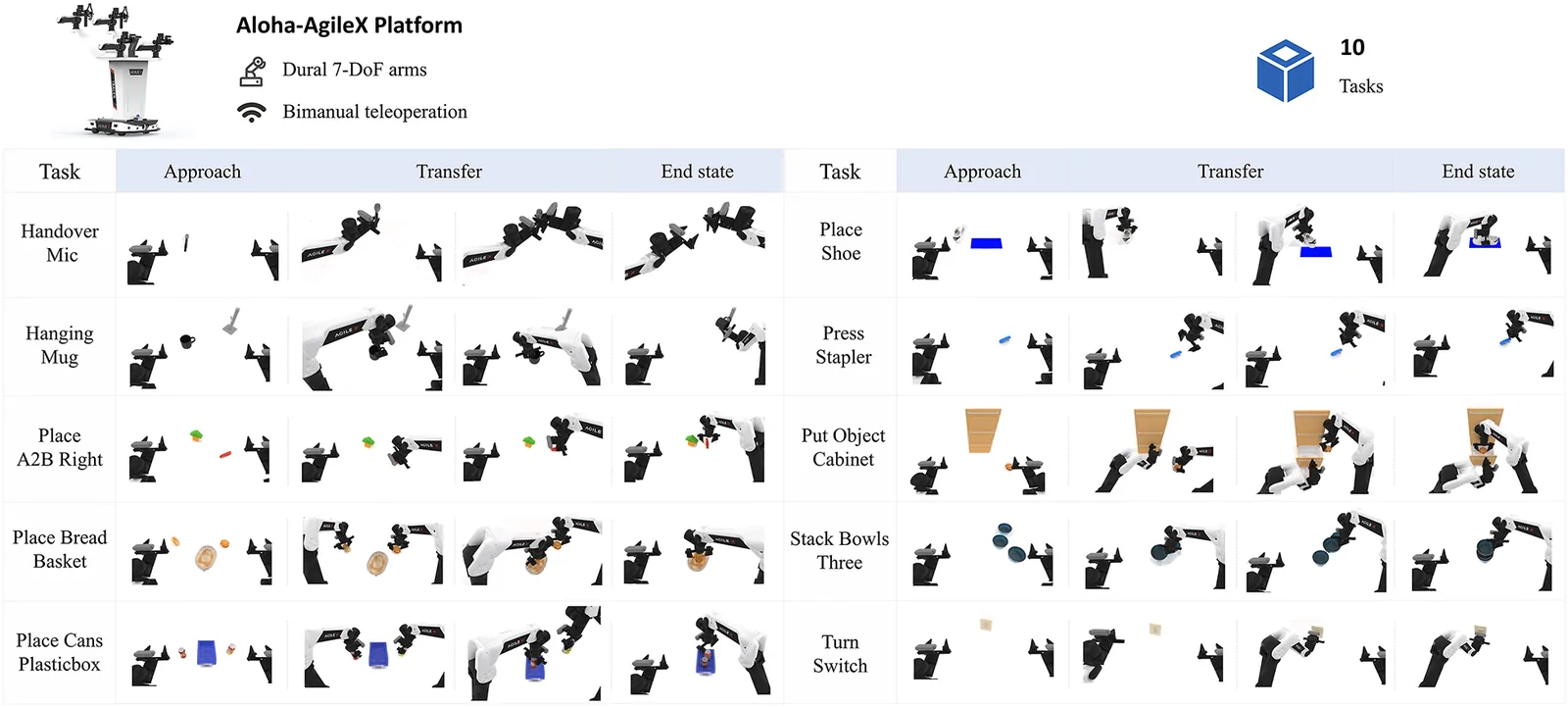
Overview of the Aloha-AgileX bimanual platform and evaluated manipulation tasks.

**Scene complexity and segmentation setup**. The main experiments follow the standard RoboTwin 2.0 benchmark with clean tabletops and task-relevant objects only. To evaluate robustness under less ideal conditions, we conduct additional experiments in Section 4.3 with distractor objects and synthetic segmentation noise.

### 4.2 Implementation details

**Evaluation protocol**. All methods are re-implemented and trained by us under identical conditions. We train a separate model for each individual task rather than a single multi-task model, following the standard practice in the RoboTwin 2.0 benchmark. For the primary experiments reported in Figures 3, 4 and throughout the main text, we conduct five independent training runs with different random seeds for each method and task, each with 100 evaluation rollouts, and report mean ± standard deviation. Error bars and ± intervals represent one standard deviation over the five runs. For the auxiliary baseline comparison in Table 1, we strictly follow the official RoboTwin 2.0 evaluation protocol with the recommended hyperparameters and settings, consistent with the published benchmark results (Chen et al., 2025). Statistical significance between our method and baselines is assessed via paired two-tailed *t*-tests (α = 0.05) based solely on the primary experimental protocol (five independent training runs with different random seeds). These statistical tests pertain exclusively to the results in Figures 3, 4 and the main text, not to the auxiliary baseline comparison in Table 1.

**Figure 3:**
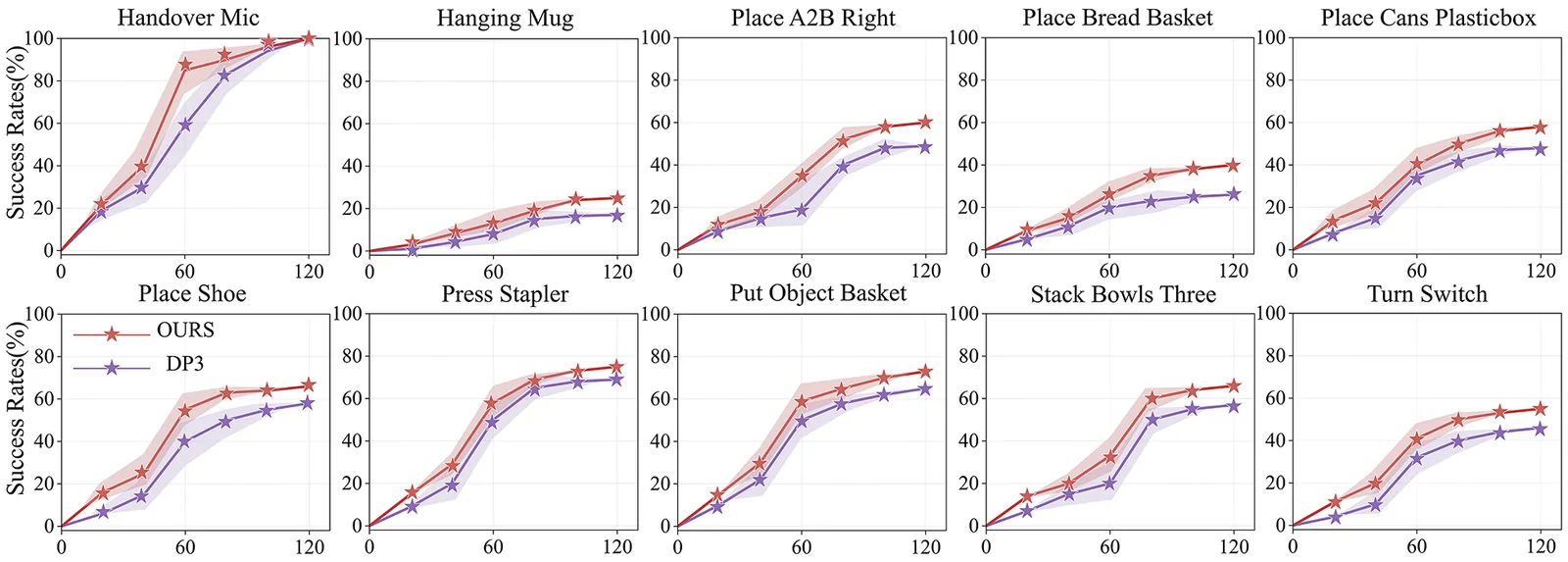
Success rate evolution across evaluated tasks.

**Figure 4:**
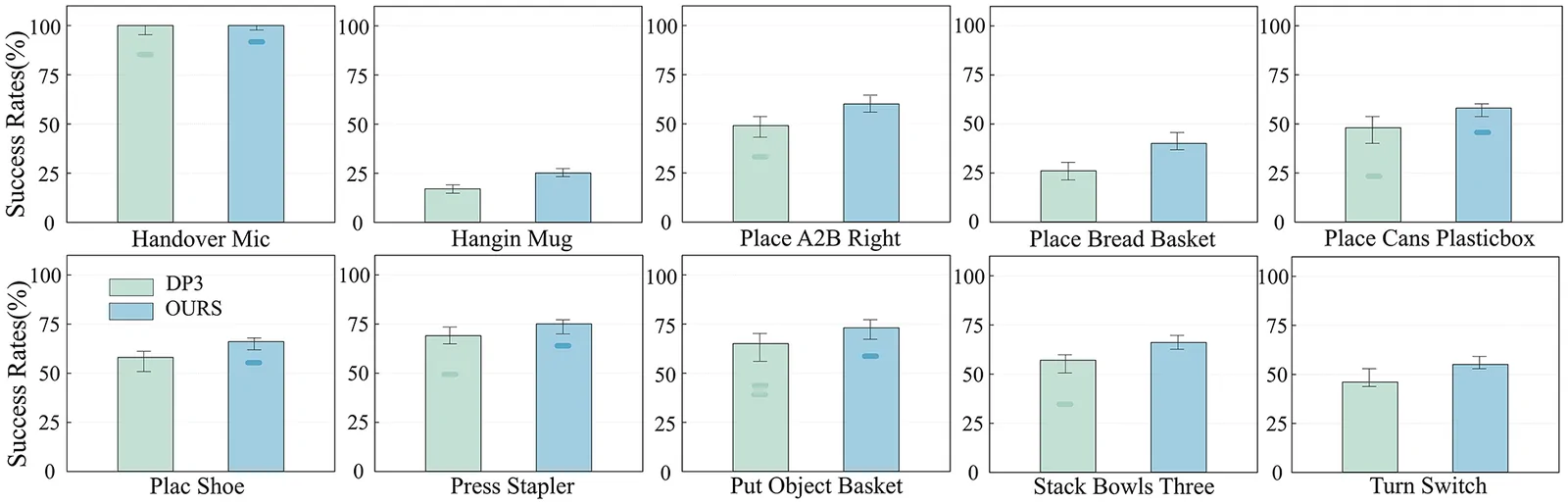
Comparative analysis of success rates across 10 evaluation tasks on the Aloha-AgileX bimanual platform.

**Table 1:** Success rate (%) comparison across methods and tasks.

Method	Handover Mic	Hanging Mug	Place A2B Right	Place Bread Basket	Place Cans Plasticbox	Place Shoe	Press Stapler	Put Object Basket	Stack Bowls Three	Turn Switch
Pi0	98% ± 1	11% ± 3	27% ± 4	17% ± 4	34% ± 6	28% ± 5	62% ± 7	16% ± 4	66% ± 6	27% ± 4
ACT	85% ± 4	7% ± 2	0% ± 0	6% ± 3	16% ± 4	5% ± 2	31% ± 6	15% ± 3	48% ± 6	5% ± 2
DP	53% ± 5	8% ± 3	13% ± 5	14% ± 3	40% ± 5	23% ± 5	6% ± 2	15% ± 4	63% ± 5	36% ± 6
DP3	100% ± 0	17% ± 4	49% ± 4	26% ± 4	48% ± 5	58% ± 6	69% ± 5	65% ± 5	57% ± 5	46% ± 5
OURS	100% ± 0	25% ± 3	60% ± 4	40% ± 3	58% ± 4	66% ± 5	75% ± 5	73% ± 4	67% ± 6	55% ± 4

All results are mean ± std over 5 independent trials with identical experimental configurations.

**Model hyperparameters**. We provide a description of the hyperparameter configurations for the core model used in our experiments. The following table summarizes the key hyperparameters optimized for our dual-arm manipulation tasks.

The prediction horizon for the Predictive Scene Module and Affordance and Coordination Evolution Module is set to *T*_*pred*_ = 4 timesteps, corresponding to 0.4 s of future states at the control frequency of 10 Hz. The historical lookback window length is *L* = 8 timesteps. The Graph Transformer employs *M* = 4 layers with 8 attention heads per layer and a hidden dimension of 256. These values are selected based on preliminary validation experiments (Table 2).

**Table 2:** Hyperparameter configuration for PACE-DP.

Hyperparameter	Value
Training	
Learning rate	1e-4
Batch size	256
Optimizer	AdamW
Weight decay	1e-6
LR scheduler	Cosine
Seed	42
Diffusion model	
Train timesteps	100
Inference steps	10
Beta range	[1e-4, 2e-2]
Model architecture	
Observation steps	3
Action steps	6
Diffusion step embed dim	64
Encoder output dim	128
Down dims	[512, 1,024, 2,048]
Kernel size	5
Point Cloud Encoder	
Point features	6
Output channels	128
PointNet type	pointnet

**Training procedure**. All modules are trained jointly end-to-end. The primary loss $L_{diff}\;$propagates gradients through **c**_*t*_ to all upstream modules. Ground-truth labels for auxiliary predictions (future scene encodings, grasp success, contact forces, relation types, coordination modes) are obtained directly from the RoboTwin 2.0 simulator via physics engine states and ground-truth object poses.

### 4.3 Experimental results

**Learning efficiency**. Throughout training, we recorded the success rate for each task to track how well our method learned and converged (Figure 3). The epoch-by-epoch results consistently show that our approach achieves faster learning and more reliable convergence in bimanual coordination settings. For each method and task, we perform 5 independent training runs with different random seeds, each consisting of 100 evaluation rollouts, and report the mean success rate.

**Computational complexity analysis**. The computational efficiency was evaluated by computing the average memory usage and inference latency of the proposed method over 10 tasks and comparing these values against the corresponding averages of the baseline methods (Table 3).

**Table 3:** Model complexity comparison.

Model	Initial memory(GB) (avg)	Peak memory (GB) (avg)	Memory increment (GB) (avg)	Avg.Inference Time (s) (avg)
DP3	2.292	2.371	0.079	1.4576
OURS	2.307	2.389	0.082	1.5748

**Task success rate evaluation**. Figure 4 reports the task success rates of all methods across the 10 evaluation tasks on the Aloha-AgileX platform. All methods are re-implemented and trained by us under identical conditions using the same simulation environment and evaluation episodes. For each method and task, we perform 5 independent training runs with different random seeds, each consisting of 100 evaluation rollouts, and report the mean success rate. Paired two-tailed *t*-tests (α = 0.05), conducted on the primary experimental results (Figures 3, 4), reveal that our method outperforms baseline methods on all tasks except the saturated “Handover Mic” (*p* < 0.05).

As shown in Table 1, we further evaluate the performance of our proposed method by comparing it with multiple baseline methods. All models are tested under identical experimental settings, including the same datasets, hardware platform, and inference conditions. For every configuration, we perform several test rounds, each comprising 100 independent inference attempts. The final success rates are obtained by averaging the results across all rounds to guarantee statistical robustness.

We evaluated whether our method depends on near-perfect segmentation by testing PACE-DP under two challenging conditions on the “Place Bread Basket” task. We introduced 3–5 distractor objects into the scene, and we corrupted instance labels with probability *p*∈{0.1, 0.2}. For each condition, we ran 5 independent trials with different random seeds, each consisting of 100 rollouts, and we report the mean success rate. As shown in Table 4, PACE-DP degrades gracefully: the success rate drops modestly in the presence of distractors, and at *p* = 0.2, performance shows only a slight decline.

**Table 4:** Success rate (%) of PACE-DP under clutter and segmentation noise on “Place Bread Basket” task (mean over 5 independent trials).

Condition	Mean
Clean	40% ± 3
With distractors (3–5 objects)	35% ± 3
Segmentation noise *p* = 0.1	37% ± 3
Segmentation noise *p* = 0.2	33% ± 4

**Ablation studies**. We evaluate each core component of PACE-DP on three representative tasks: “Place Cans Plasticbox,” “Place Bread Basket,” and “Stack Bowls Three.” The following variants are compared:

- **w/o PSM:** Excludes predicted future representations ${\hat{H}}_{t+1:t+T_{pred}}^{pred}$ from conditioning.
- **w/o AREM:** Removes relation embeddings $E_{t}^{rel}$ and coordination embedding $z_{t}^{coord}$; disables Graph Transformer and coordination submodule.
- **w/o CoordPred:** Removes coordination classifier and loss $L_{coord}$.
- **w/o CoordModule:** Removes the entire coordination submodule, including both the coordination embedding and the coordination classifier.
- **w/o AffordLoss:** Excludes affordance grounding loss $L_{afford}$.
- **w/o SmoothLoss:** Excludes temporal smoothness loss $L_{smooth}$.
- **Full Model:** All components enabled.

As shown in Table 5, removing the Affordance and Coordination Evolution Module (w/o AREM) causes the largest performance drop (14%−17%), confirming that modeling dynamic relationship evolution is most critical for complex bimanual tasks. Removing the entire coordination submodule (w/o CoordModule) leads to a 10%−13% drop, notably larger than removing only the coordination classifier (w/o CoordPred, 4%−7%), indicating that both the coordination representation and its predictive supervision contribute meaningfully. Excluding individual loss terms (w/o AffordLoss, w/o SmoothLoss) results in smaller drops of 2%−5%. These results confirm that each proposed component positively contributes to the final performance.

**Table 5:** Ablation study results: success rate (%) on three representative tasks (mean ± std over 5 runs).

Variant	Place Cans Plasticbox	Place Bread Basket	Stack Bowls Three
w/o PSM	50% ± 4	31% ± 4	58% ± 6
w/o AREM	44% ± 5	26% ± 4	50% ± 5
w/o CoordPred	54% ± 4	34% ± 4	60% ± 5
w/o CoordModule	48% ± 5	28% ± 4	54% ± 6
w/o AffordLoss	55% ± 4	35% ± 3	62% ± 6
w/o SmoothLoss	56% ± 4	36% ± 4	63% ± 7
Full Model	58% ± 4	40% ± 3	67% ± 6

## 5 Conclusion

This work introduced Predictive Affordance and Coordination Evolution Diffusion Policy (PACE-DP), a novel diffusion-based framework for bimanual manipulation that integrates future scene prediction, affordance evolution modeling, and coordination dynamics into a unified action generation pipeline. By leveraging a predictive scene module and a Graph Transformer architecture to capture temporal relationship changes, PACE-DP generates smooth, coordinated action sequences conditioned on fused current and forecasted features. Extensive experiments on the RoboTwin 2.0 platform across 10 bimanual tasks demonstrate that our approach consistently outperforms the baseline method, achieving higher success rate improvements on challenging tasks while incurring marginal computational overhead.

**Limitations**. Several limitations of the current work should be acknowledged. The method has been evaluated exclusively in simulation under relatively clean scenes. Although our robustness analysis shows resilience to distractors and segmentation noise, physical hardware validation in highly cluttered environments remains necessary. The coordination classifier is supervised from demonstrations, limiting adaptability to unseen patterns, and the predictive module assumes consistent object tracking. Future work will address sim-to-real transfer, online segmentation, and self-supervised coordination learning.

## Data Availability

The data used in this study were generated using the RoboTwin 2.0 simulation platform through its automated task execution pipeline. The datasets consist of expert demonstration trajectories synthesized under domain-randomized settings and were generated specifically for the experiments reported in this paper. Due to storage and platform constraints, the datasets are not publicly hosted. However, the data are available from the authors upon reasonable request for research and verification purposes. Requests to access the datasets should be directed to 2898627792@qq.com.
